# Triple Valve Endocarditis and Multiorgan Failure: A Clinical Conundrum

**DOI:** 10.7759/cureus.81603

**Published:** 2025-04-02

**Authors:** Amey Joshi, Sarah P Alizadeh, Harith Ghnaima, Zan Siddiqi, Selwan Edward

**Affiliations:** 1 Internal Medicine, Michigan State University, Lansing, USA; 2 Internal Medicine, Michigan State University College of Human Medicine, Lansing, USA; 3 Critical Care Medicine, Sparrow Hospital, Lansing, USA

**Keywords:** infective endocarditis, methicillin resistant staphylococcus aureus (mrsa), renal failure, septic shock, triple valve endocarditis

## Abstract

Triple valve infective endocarditis is a rare clinical diagnosis with limited evidence on treatment strategies. Complications, including congestive heart failure, renal failure, and persistent bacteremia, predispose to increased mortality risk and limit surgical options. Due to its rarity, little is known about its prognosis and best treatment strategies. We present a case of a young man with multiple comorbidities, including liver cirrhosis and paroxysmal atrial fibrillation, who was diagnosed with right-sided infective endocarditis. Due to the patient’s complex clinical course, his admission was complicated with infective endocarditis of the tricuspid valve, mitral valve, and aortic valve. Alongside its rarity, this case highlights the limited interventions at our disposal in treating triple valve endocarditis with multiple high-risk factors.

## Introduction

Triple valve infective endocarditis (TVE) is a rare diagnosis, with only a handful of cases being reported. Risk factors for TVE include intravenous (IV) drug use, preexisting valvular disease, hemodialysis, the presence of intracardiac devices, diabetes mellitus, and immunosuppression [1]. Due to its rarity, little is known about its prognosis and best treatment strategies. Despite recent advancements in operative techniques, the management of multi-valve endocarditis (MVE) remains challenging. We present a case of a young male with a complex medical history whose clinical course led to triple valve IE. This case also highlights the limited medical and surgical options in triple valve IE in the setting of multiple high-risk factors.

## Case presentation

A 37-year-old male with a past medical history of alcohol use, liver cirrhosis, and paroxysmal atrial fibrillation presented with lethargy and fatigue. He had a recent hospital admission one month prior to presentation when he was treated for methicillin-resistant Staphylococcus aureus (MRSA) bacteremia from lower limb cellulitis. He received IV antibiotics for 14 days, which included IV vancomycin and IV metronidazole. An echocardiogram that was done during that admission did not show any evidence of valvular vegetation.

On presentation, he was noted to be acutely confused, hypotensive, not responding to fluids, tachypneic, and in atrial fibrillation with rates of 160 beats/minute. Physical examination was noteworthy for splenomegaly, Osler’s nodes, and Janeway lesions.

His laboratory investigations were significant for lactic acidosis and increased pro-inflammatory markers, including procalcitonin and C-reactive peptide, anemia (hemoglobin 7.0 g/dl), and thrombocytopenia (48,000/mm^3^) (Table 1).

**Table 1:** Laboratory values on admission AST: aspartate transaminase; ALT: alanine transaminase; BUN: blood urea nitrogen, aPTT: activated partial thromboplastin time, INR: international normalized ratio, WBC: white blood cell count

Lab Test	Admission Laboratory Values	Reference Ranges and Units
Troponin I High Sensitivity (ng/L)	2,268	0-18
B-Natriuretic Peptide (pg/mL)	407	0-100
Sodium (meq/L)	126	135-145
Potassium (meq/L)	5.9	3.5-4.9
BUN (mg/dL)	30	6-23
Creatinine (mg/dL)	1.02	0.60-1.40
Calcium (mg/dL)	7.19	8.00-10.50
Albumin (g/dL)	2.5	3.6-5.0
AST (U/L)	33	10-40
ALT (U/L)	9	3-45
Alkaline Phosphatase (U/L)	144	45-115
Bilirubin, Total (mg/dL)	1.9	0.2-1.2
C-Reactive Protein (mg/dL)	12.6	0.0-1.0
Lactate (mmol/L)	7.2	0.2-1.8
Procalcitonin (ng/mL)	1.75	0.0-0.09
aPTT (seconds)	54.9	21.0-31.0
Prothrombin Time (seconds)	17.8	9.5-12.1
INR	1.7	2.0-3.0
WBC (x 10^3 ^/µL)	18.9	4.0-12.0
Hemoglobin (g/dL)	8.4	12.6-16.5
Platelet Count (x 10^3 ^/µL)	68	150-450
Neutrophils	96%	49.0-81.0%
Absolute Neutrophils (x 10^3 ^/µL)	18.14	1.96-9.72
Iron, Total (µg/dL)	11	65-150
Total Iron Binding Capacity (µg/dL)	174	270-440
Iron Saturation	6%	20.0-50.0%
Sedimentation Rate (mm/hr)	34	0-15

**Figure 1:**
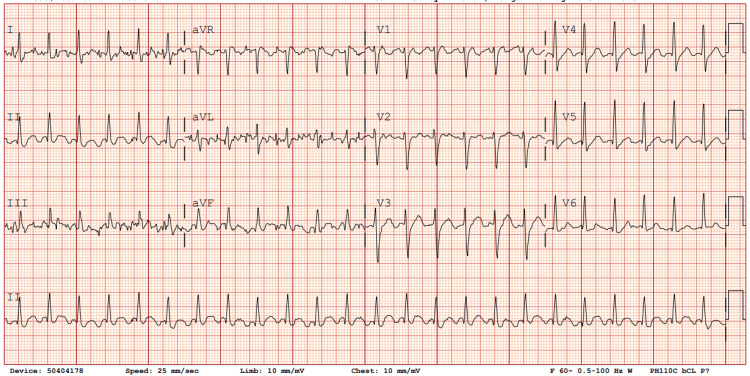
EKG showing atrial flutter

**Figure 2:**
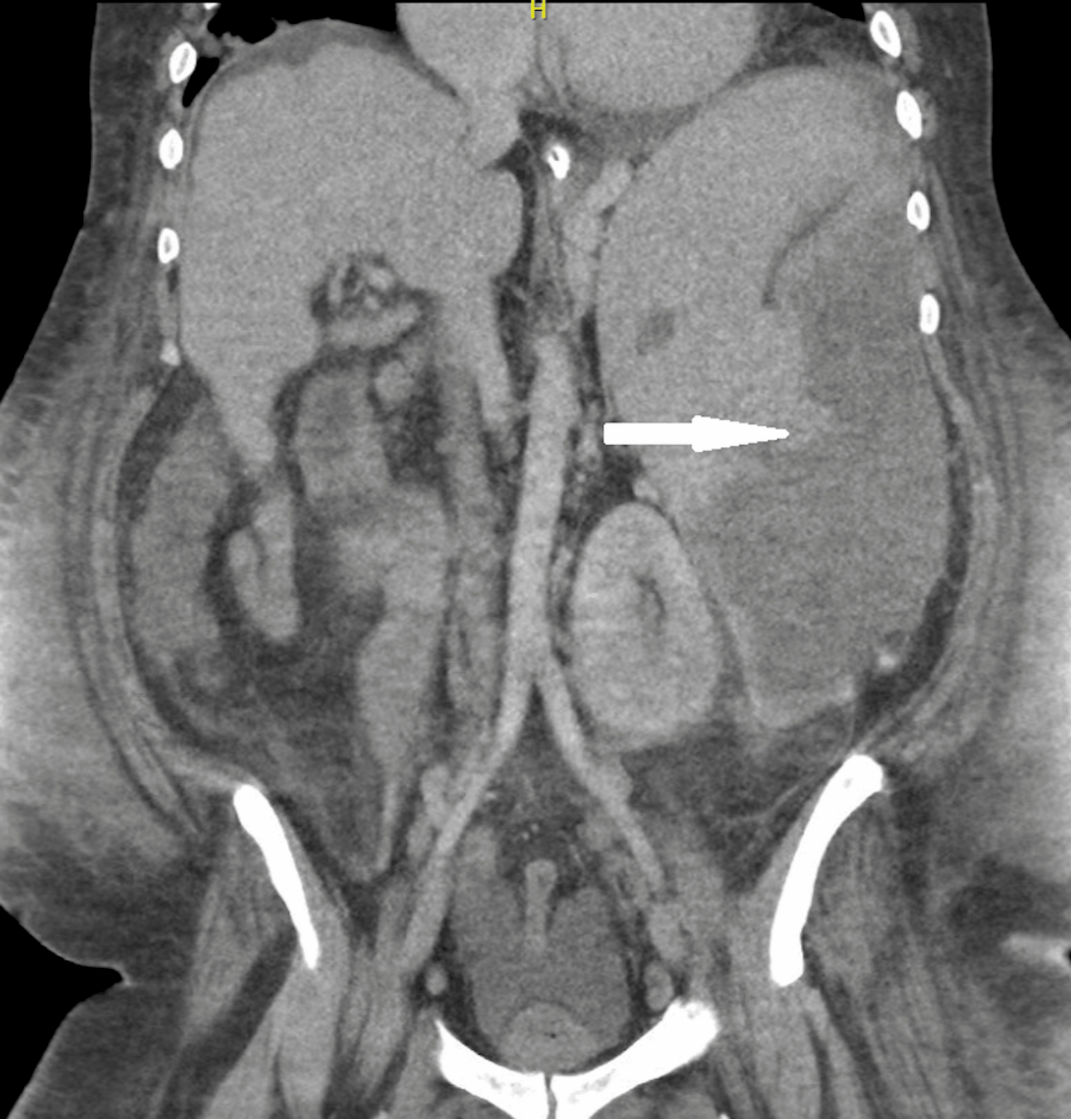
CT abdomen showing splenic infarct (white arrow)

**Figure 3:**
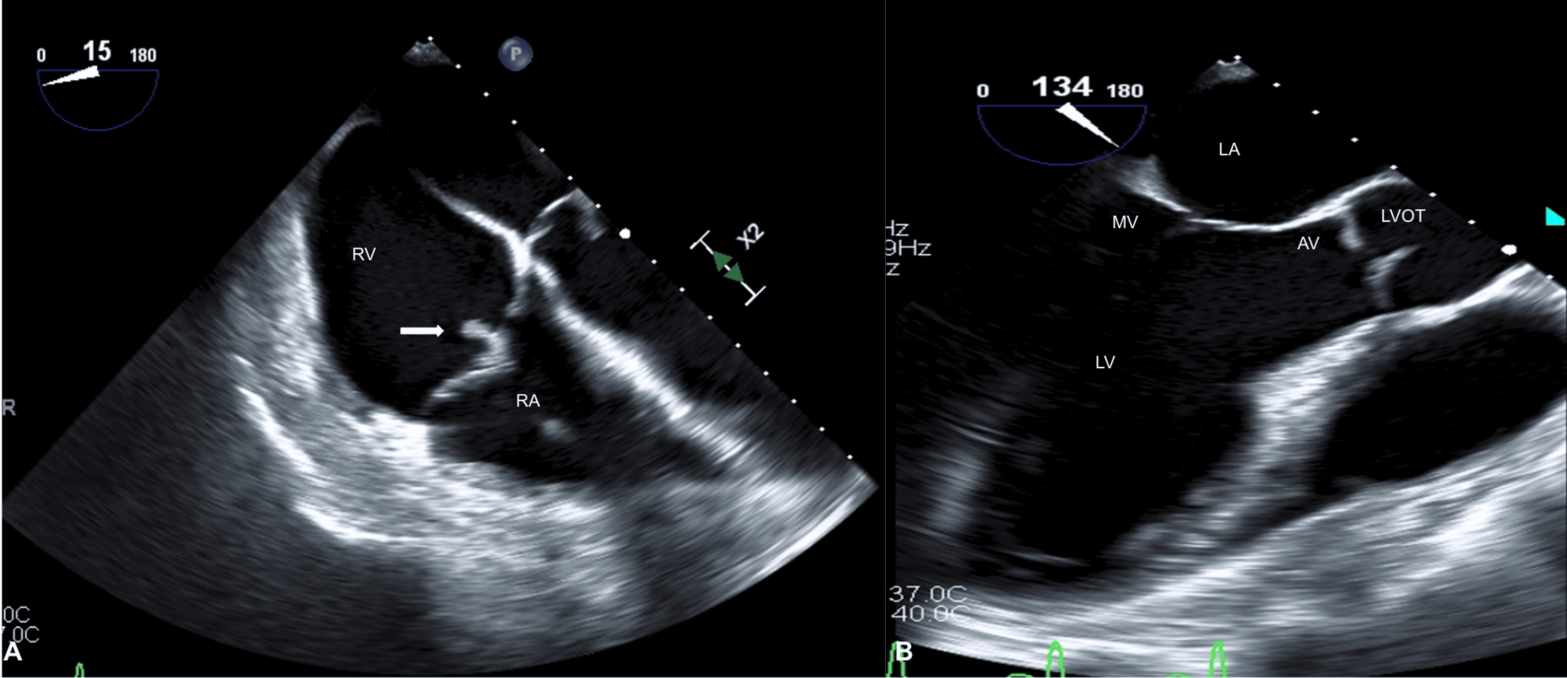
Transesophageal echocardiogram showing A) mobile echodensity on tricuspid valve measuring 0.7 x 0.7 cm (white arrow); B) normal mitral valve and aortic valve without vegetations RV: right ventricle, RA: right atrium, LA: left atrium, MV: mitral valve, LV: left ventricle, AV: aortic valve, LVOT: left ventricular outflow tract

EKG confirmed atrial flutter (Figure 1). CT imaging of the chest and abdomen revealed multiple nodules with cavitations in the lung and a splenic infarct (Figure 2). Blood cultures were positive for MRSA. An initial transesophageal echocardiogram (TEE) revealed a mobile echodensity on the tricuspid valve measuring 0.7 x 0.7 cm (Figure 3).

**Figure 4:**
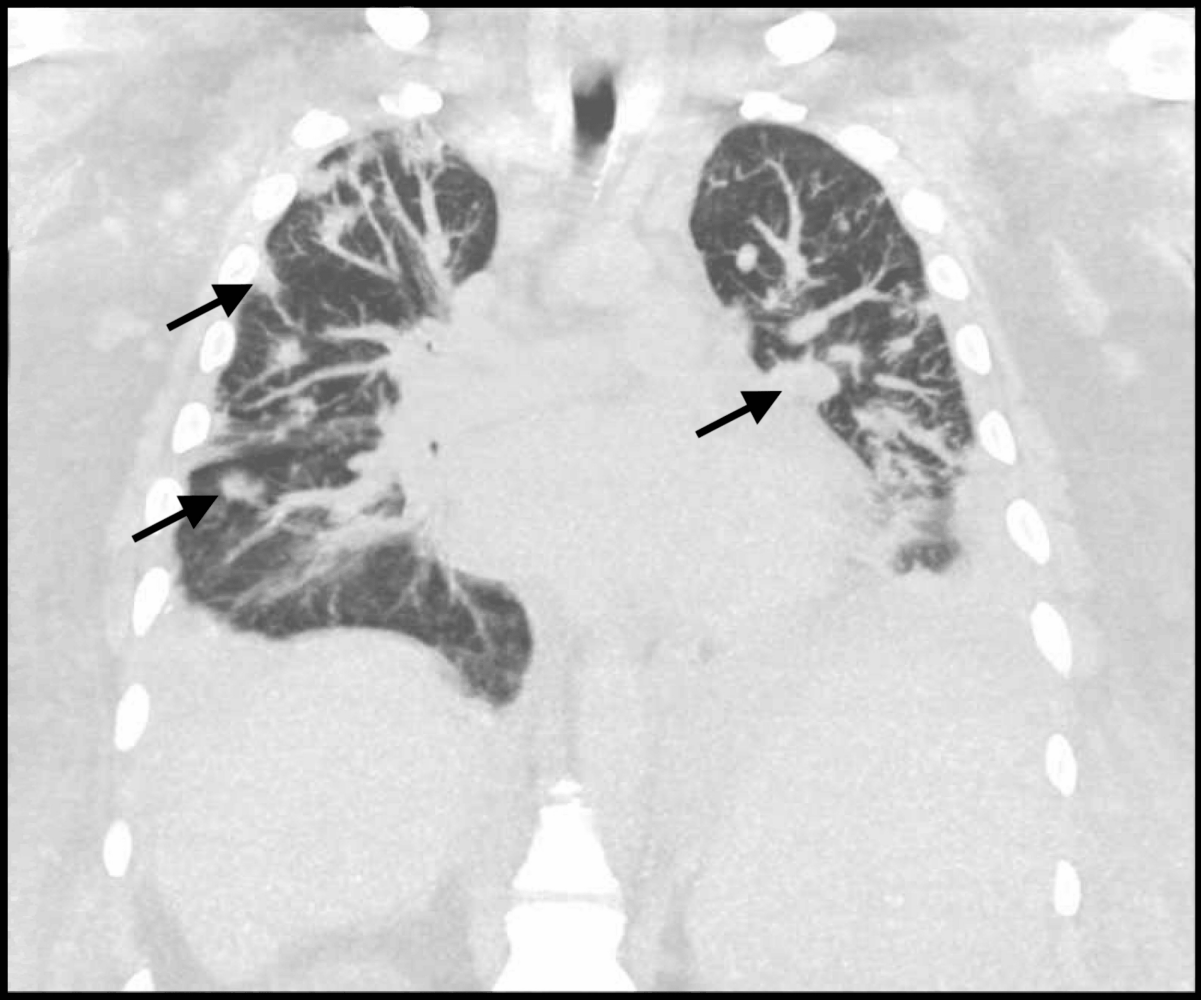
CT lung showing multinodular opacities suggestive of necrotizing pneumonia and abscess formation (black arrows)

The patient was started on vancomycin; however, due to acute kidney injury, antibiotics were changed to daptomycin and ceftaroline for the right-sided infective endocarditis complicated by pulmonary septic emboli. For the concern of hemodynamically unstable atrial flutter, despite digoxin and vasopressor support, the decision was taken to cardiovert the patient; however, this was unsuccessful. Subsequently, the patient’s clinical course was complicated by the development of a large, left-sided empyema requiring chest tube insertion (Figure 4).

**Figure 5:**
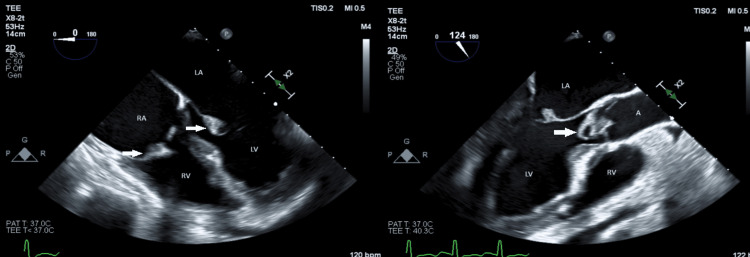
Transesophageal echocardiography showing vegetations (white arrows) in tricuspid valve, mitral valve, and aortic valve RA: right atrium, LA: left atrium, RV: right ventricle, LV: left ventricle, A: aorta

Pleural fluid cultures were also positive for MRSA. Due to persisting bacteremia, the patient underwent a repeat TEE that was remarkable for new vegetations on the aortic valve (1.89 x 1.21 cm) and mitral valve (1.43 x 0.68 cm) and an increase in the size of the tricuspid vegetation (2 x 2.7 cm) (Figure 5).

Surgical valve repair was deemed extremely high risk, and the decision was made to continue medical management. Despite aggressive medical management of MRSA sepsis, atrial fibrillation, heart failure, and chest tube placement for empyema, the patient was intubated and started on continuous renal replacement therapy but unfortunately passed away from multiorgan failure.

## Discussion

TVE presents unique challenges and represents a separate clinical entity due to being recognized as an independent risk factor affecting mortality. The condition’s rarity adds to its complexity due to the limited evidence on optimal management techniques. One study reported the incidence of multivalve endocarditis to be nearly 18%; however, none of the cases studied involved TVE. A recent review in 2018 reported only 16 published case reports of TVE [2]. Common risk factors for TVE include IV drug use, pre-existing valvular disease, diabetes mellitus, and immunosuppression [1]. In the present case, the patient’s liver disease predisposed him to being immunosuppressed, and his recent history of MRSA bacteremia was likely the inciting factor for the IE.

Emboli to the brain, lungs, or spleen occur in nearly 30% of patients. Our patient’s clinical course was complicated by multiple septic pulmonary emboli and splenic abscesses [3]. Besides valvular complications, IE can be complicated by multiorgan failure and septic shock in the setting of persisting bacteremia [4]. Transesophageal echocardiography (TEE) remains the gold standard for detecting valvular vegetation, with sensitivity and specificity ranging from 87-100% and 91-100%, respectively. However, TTE may help detect right-sided vegetation due to its proximity to the chest wall. Three-dimensional (3D) and 2D TEE have reported mixed evidence of superiority with comparable sensitivities [5].

Patients with tricuspid valve endocarditis can be successfully treated with antibiotics, and surgery is reserved for those with large vegetations, recurrent septic pulmonary emboli, failure of medical therapy, infected prosthetic valves, and heart failure [6]. Right-sided IE with concomitant left-sided IE carries a significantly worse prognosis due to the predilection for invasion and abscess formation [7,8]. A recent study found mortality rates to increase up to 4.7 times with two-valve IE and up to 14 times with triple-valve IE when compared to single-valve disease [2,9-11]. Surgical intervention is vital in managing MVE and is associated with improved survival [3]. Early and late perioperative mortality rates, however, are higher in multivalve disease repair compared to single valve disease and can range up to 25% and 31%, respectively [1]. Concomitant renal failure and congestive heart failure can also significantly increase perioperative mortality [10,11]. Persistent infection was also shown to be associated with an increased risk of in-hospital mortality of up to four-fold [11]. Different scoring systems, including the Risk-E score, have been employed to predict perioperative mortality in IE [12]. In the present case, the Risk-E score predicted perioperative mortality to be nearly 72.32%, and surgery was deferred.

## Conclusions

TVE is a rare clinical entity that poses significant challenges in management. Multivalvular involvement, congestive heart failure, renal failure, and persisting bacteremia are significant risk factors that increase perioperative mortality. Surgery should be considered early in cases of TVE before the development of complications. However, by the time the diagnosis of TVE is established, these complications may have already developed, which significantly impede surgical options. Due to the limited evidence, there is a lack of clear guidelines and optimal therapeutic strategies in TVE.
